# Epidemiology of Paediatric Trauma During National Lockdown: A Retrospective Study With 12 Months of Follow-Up

**DOI:** 10.7759/cureus.47855

**Published:** 2023-10-28

**Authors:** Catherine Qin, Rupen Tamang, Dominic Waugh, James Grayston, Mohammad Al-Ashqar, Peyman Bakhshayesh, Laura Deriu

**Affiliations:** 1 Department of Accident and Emergency, The Royal London Hospital, Barts Health NHS Trust, London, GBR; 2 Department of Trauma and Orthopaedics, Leeds Teaching Hospitals NHS Trust, Leeds, GBR; 3 Department of Trauma and Orthopaedics, University Hospital Crosshouse, Kilmarnock, GBR

**Keywords:** covid-19, school closure, injury, paediatric, trauma, epidemiology, lockdown

## Abstract

Introduction

The COVID-19 pandemic and its associated preventative measures such as national lockdown dramatically changed the daily activities of children. This paper aims to compare the epidemiology of paediatric orthopaedic trauma presentation, management and outcomes during the school closure period with the matched pre-pandemic period in 2019.

Methods

This was a retrospective cohort study of data collected from the West Yorkshire Trauma Network, comprising a major trauma centre, Leeds General Infirmary, and five peripheral trauma units. All patients aged 0-18 years who required trauma unit management during the school closure period (18 March 2020-25 May 2020) were included. Cases for the matched period in 2019 were analysed for baseline comparison. Patient demographics, mechanism and anatomical location of injury, management and follow-up were assessed.

Results

In the 2020 and 2019 cohorts, 286 and 575 injuries were observed, respectively. In the 2020 cohort, we observed a 50.3% (n=289) fall in paediatric trauma presentation and a significant proportional reduction in referrals from the emergency department (22% (n=63) versus 53% (n=305); p<0.001). There was also a significant reduction in the average age at presentation by more than one year (p<0.001). Sports-related injuries decreased significantly (n=16 (5.6%) versus n=127 (22.1%); p<0.001). While the proportion of ride-on injuries increased significantly, overall numbers remained similar (n=63 (22%) versus n=61 (10.6%); p<0.0001). Non-accidental injury (NAI) concerns rose significantly (n=9 (3.1%) versus n=4 (0.7%); p=0.01), but the absolute number of confirmed NAI cases stayed the same (n=2). There was a proportional increase in upper limb injuries (64.3% (n=184) versus 58.4% (n=336); p>0.05) and a proportional reduction in lower limb injuries (32.1% (n=92) versus 35.5% (n=204); p>0.05). However, the rate of tibial shaft injuries rose significantly (10.1% (n=29) versus 5.2% (n=30); p=0.02). The use of conservative management increased with a significant delay in average time to surgery from the date of injury (8.5 days versus 3.1 days; p=0.01). Patients who were only followed up with a telephone consultation rose significantly (23% (n= 66) versus 6% (n=35); p<0.001). Re-presentation rate increased significantly (1.4% (n=4) versus 0.2% (n=12); p=0.04).

Conclusion

Our study showed a reduction in paediatric trauma presentations during the pandemic and a significant reduction in the average age at presentation. This change has been accompanied by a shift in the mechanism and anatomical location of injury, management and subsequent follow-up.

## Introduction

The COVID-19 pandemic led to widespread changes in British society. Measures to prevent the spread of COVID-19 included social distancing, school closures and sports cancellation as they were shown to be effective in the past [1,2]. In March 2020, the prime minister announced a strict national lockdown [3]. On 18 March 2020, all schools in the United Kingdom were shut down [3]. These policies inevitably changed the daily lives of children and their activities. These changes may have altered the epidemiology of paediatric trauma presentation during the COVID-19 pandemic. Lockdown measures may also have affected management and outcomes. A better understanding of epidemiological trends can lead to improved injury prevention, resource utilisation, access to care and clinical outcomes.

The aim of this paper is to understand the effect that the school closure had on paediatric trauma services within the region of West Yorkshire. This includes a review of follow-up in the subsequent 12-month period to determine clinical efficacy, safety and an optimal strategy for future service allocation.

This article was previously presented as a poster at the European Federation of National Associations of Orthopaedics and Traumatology on 22 June 2022. In addition, it was posted to the Authorea preprint server on 27 September 2023.

## Materials and methods

This institutional review board-approved retrospective cohort study comprises paediatric trauma patients of the West Yorkshire Trauma Network. This network is coordinated by Leeds General Infirmary, a major paediatric trauma centre, supported by five peripheral trauma units. Included in the study were paediatric trauma patients who were managed within two time periods: lockdown, defined as the duration of national school closure (18 March 2020-25 May 2020), and a matched period in 2019 to act as a baseline for comparison.

Patients were identified through electronic trauma handover lists, hospital admissions, theatre lists and fracture clinic lists. Included were all patients under the age of 18 years who were managed by the trauma and orthopaedics department during our study periods. Exclusion criteria were duplicates, non-trauma presentations and patients managed by the minor injuries unit or other departments.

Patient demographics including age at presentation, sex and comorbidities were recorded, as well as the source of referral. Table 1 summarises data that were collected on anatomical location, type of injury, mechanism of injury and management. A review of follow-up in the subsequent 12-month period was carried out, and any complications or adverse outcomes were recorded.

**Table 1:** Data collected on anatomical location, type of injury, mechanism of injury and management

Data collection		
Anatomical location of injury	Upper limbs	Hand
		Wrist
		Forearm
		Elbow
		Supracondylar
		Humeral shaft
		Shoulder
		Clavicle
	Lower limbs	Foot
		Ankle
		Tibial shaft
		Knee
		Femur
		Pelvis
	Axial skeleton	Head
		Spine
	Polytrauma	
	Others	
Type of injury	Nil	
	Sprains and blunt trauma	
	Wound	
	Dislocation	
	Fracture	
	Open injury	
Mechanism of injury	Normal fall: falling while walking/running/playing on level ground	
	Great fall: falling from a height of over 1 meter	
	Ride-on injury: bikes/scooters/skates, etc.	
	Outdoor toys: trampolines/swings/slides, etc.	
	Sports-related injury	
	Road traffic collision	
	Non-accidental injury	
	Others	
Management	Conservative: reassurance/physiotherapy/analgesia	
	Sling/splint/boot	
	Back-slab/cast	
	Manipulation +/- cast	
	Manipulation + K-wires	
	Intramedullary nails	
	Open reduction internal fixation/plates and screws	
	External fixation	
	Wound surgical management: washout/debridement/closure/foreign body removal	
	Other specialty care	

The chi-square test was utilised to evaluate the effect of lockdown on categorical variables, while the Mann-Whitney U test was used for continuous variables. In all cases, statistical significance was set at p<0.05.

## Results

Demographics

In 2020, there were a total of 286 paediatric trauma cases, which was a 50.3% (n=289) fall from 575 cases in 2019. A significant difference in the average age of patients at presentation between the two periods was observed. During the school closure period, the mean age was 8.3 years, whereas in 2019, it was 9.5 years (p<0.001). The ratio between male and female patients remained similar in both time periods (1:0.673 in 2020 versus 1:0.634 in 2019; p>0.05). There was a significant reduction in patients with a comorbidity that was likely to affect management decisions during the 2020 period (n=4 (1.4%) versus n=43 (7.5%); p<0.001). The most common comorbid conditions across both periods were asthma, developmental delay and hypermobility.

Source of referral

Table 2 summarises the source of referral. Out of the 286 patients seen in 2020, 22% (n=63) presented directly to the emergency department. This was a significant reduction in proportion compared to 2019 when 53% (n=71) of all paediatric referrals (n=575) were from the emergency department (p<0.001).

**Table 2:** Source of referral

Source of referral	Number and percentage in 2019 (n=575)	Number and percentage in 2020 (n=286)	P-values
Virtual fracture clinic	407 (71%)	220 (77%)	>0.05
On-call team	134 (23%)	63 (22%)	>0.05
Tertiary centre	34 (6%)	3 (1%)	>0.05

Mechanisms

Table 3 summarises the mechanism of injury. Although normal falls accounted for the largest proportion of injuries in both time periods, they accounted for a greater proportion of injuries in 2020. Despite the proportional increase, the overall numbers decreased (n=129 (45.1%) versus n=203 (35.3%); p>0.05). Compared to 2019, there was a significant reduction in sports-related injuries during the school closure period (n=16 (5.6%) versus n=127 (22.1%); p<0.001). A significant proportional increase in ride-on injuries was observed in 2020, although overall numbers remained similar (n=63 (22%) versus n=61 (10.6%); p<0.0001). Non-accidental injury (NAI) concerns also rose significantly in 2020 (n=9 (3.1%) versus n=4 (0.7%); p=0.01). However, the absolute number of confirmed NAI cases stayed the same for both periods (n=2).

**Table 3:** Mechanism of injury

Mechanism	Number and percentage of injuries in 2019 (n=575)	Number and percentage of injuries during school closure in 2020 (n=286)	P-values
Normal falls	203 (35.3%)	129 (45.1%)	>0.05
Great falls	32 (5.6%)	13 (4.5%)	>0.05
Sports-related injury	127 (22.1%)	16 (5.6%)	<0.001
Ride-on injury	61 (10.6%)	63 (22%)	<0.0001
Outdoor toys	102 (17.7%)	52 (18.2%)	>0.05
Road traffic collision	24 (4.2%)	6 (2.1%)	>0.05
Non-accidental injury concerns	4 (0.7%)	9 (3.1%)	0.01
Confirmed non-accidental injury cases	2 (0.3%)	2 (0.7%)	>0.05
Others	20 (3.5%)	5 (1.7%)	>0.05
Unreported	4 (0.7%)	0 (0%)	>0.05

Injury location

Table 4 summarises the anatomical location of injury. There was a proportional increase in upper limb injuries during the school closure period (n=184 (64.3%) versus n=336 (58.4%); p>0.05). Specifically, the rate of injuries to wrists, forearms, elbows and supracondylar regions increased. Although there was a proportional reduction across all lower limb injuries in 2020 (n=92 (32.1%) versus n=204 (35.5%); p>0.05), tibial shaft injury rates rose significantly (n=29 (10.1%) versus n=30 (5.2%); p=0.02).

**Table 4:** Anatomical location of injury

Anatomical location		Number and percentage of injuries in 2019 (n=575)	Number of percentage of injuries during school closure in 2020 (n=286)	P-values
Upper limbs	Hand	8 (1.4%)	2 (0.7%)	>0.05
	Wrist	128 (22.3%)	81 (28.3%)	>0.05
	Forearm	30 (5.2%)	22 (7.7%)	>0.05
	Elbow	78 (13.6%)	39 (13.6%)	>0.05
	Supracondylar	46 (8%)	29 (10.1%)	>0.05
	Humeral shaft	4 (0.7%)	1 (0.3%)	>0.05
	Shoulder	16 (2.7%)	2 (0.7%)	>0.05
	Clavicle	26 (4.5%)	8 (2.8%)	>0.05
Lower limbs	Foot	64 (11.1%)	28 (9.8%)	>0.05
	Ankle	61 (10.6%)	21 (7.3%)	>0.05
	Tibial shaft	30 (5.2%)	29 (10.1%)	0.02
	Knee	33 (5.7%)	8 (2.8%)	>0.05
	Femur	13 (2.3%)	5 (1.7%)	>0.05
	Pelvis	3 (0.5%)	1 (0.3%)	>0.05
Axial skeleton		13 (2.3%)	5 (1.7%)	>0.05
Polytrauma		5 (0.9%)	3 (1%)	>0.05
Other		17 (3%)	2 (0.7%)	>0.05

Injury type

Table 5 summarises the types of injury. There was a proportional increase in fractures during the school closure period (n=228 (79.7%) versus n=404 (70.3%); p>0.05), and this was the most common type of injury in both time periods. The proportion and absolute number of open injuries increased significantly (n=10 (3.5%) versus n=7 (1.2%); p=0.035).

**Table 5:** Type of injury

Type of injury	Number and percentage of injuries in 2019 (n = 575)	Number and percentage of injuries during school closure in 2020 (n = 286)	P-values
Fracture	404 (70.3%)	228 (79.7%)	>0.05
Sprains and blunt trauma	116 (20.2%)	39 (13.6%)	>0.05
Wounds	14 (2.4%)	5 (1.7%)	>0.05
Dislocations	12 (2.1%)	4 (1.4%)	>0.05
Open injury	7 (1.2%)	10 (3.5%)	0.035
Nil	14 (2.4%)	2 (0.7%)	>0.05

The number of injuries that were associated with neurovascular compromise decreased in 2020 (n=13 (2.3%) versus n=4 (1.4%); p>0.05).

Management

Table 6 summarises management and interventions. The use of a back-slab or cast was the most common intervention in both periods. Operative rates were lower in the 2020 period (n=27 (9.4%) versus n=80 (13.9%); p>0.05). Additionally, there was a proportional increase in the use of conservative management or simple interventions during the school closure period (n=234 (81.8%) versus n=411 (71.5%); p>0.05). A significant increase in the average time to surgery from the date of injury was observed (8.5 days versus 3.1 days; p=0.01). The mean length of inpatient stay reduced significantly from 0.7 days in 2019 to 0.4 days (p=0.004).

**Table 6:** Management and intervention ORIF: open reduction internal fixation

Management	Number and percentage of injuries in 2019 (n=575)	Number and percentage of injuries during school closure in 2020 (n=286)	P-values
Reassurance/physiotherapy/analgesia	65 (11.3%)	19 (6.6%)	>0.05
Back-slab/cast	221 (38.4%)	124 (43.4%)	>0.05
Sling/splint/boot	152 (26.4%)	87 (30.4%)	>0.05
Manipulation + cast	38 (6.6%)	24 (8.4%)	>0.05
Manipulation + K-wires	24 (4.2%)	5 (1.7%)	>0.05
Wound surgical management	15 (2.6%)	8 (2.8%)	>0.05
ORIF/plates and screws	30 (5.2%)	9 (3.1%)	>0.05
External fixation/frames	0 (0%)	0 (0%)	>0.05
Intramedullary nails	11 (1.9%)	5 (1.7%)	>0.05
Other specialty care	18 (3.1%)	5 (1.7%)	>0.05

Follow-up and complications

The use of telephone consultation for follow-up increased significantly (n=67 (23%) versus n=36 (6%); p<0.001). Table 7 summarises a list of complications across both periods. The rate of complications from management increased in the school closure period (n=7 (2.4%) versus n=9 (1.6%); p>0.05). Re-presentation increased significantly in 2020 (n=4 (1.4%) versus n=1 (0.2%); p=0.04). However, out of all the cases in the 2020 period, only one case required additional surgical management (n=1 (25%)). No deaths were recorded in either group.

**Table 7:** Complications from interventions and management TENS: titanium elastic nail system, OK: Outerbridge-Kashiwagi

List of complications in 2019	List of complications in 2020
Failed K-wires that needed re-operation	Malunion - referred for osteotomy
Mild forearm deformity that was accepted	Varus deformity, slow progress - referred for osteotomy
Knee pain after removal of femoral TENS nails	2 cases of mild forearm deformity that was accepted
Rotational mal-alignment	Irritation from metal work on tendons - referred for removal
Malunion and reduced range of movement	Elbow extension movement blocked - underwent reoperation for removal of screw and OK procedure
Anterior knee pain for removal of metal after tibial intramedullary nail	
Prolonged pain issues - referred to adult knee surgeon	
Prominent titanium elastic nail	
Needed further terminalisations (amputations)	

## Discussion

Lockdown measures, in response to the COVID-19 pandemic, had a profound effect on all aspects of daily life for children. This study examined the epidemiological trends in paediatric trauma presentation across the West Yorkshire Trauma Network during the school closure period. This demonstrated a reduction in the overall volume of injuries and decreased mean patient age. Additionally, there was a shift in the distribution of injury types and changes in management and follow-up.

Presentation

Our results showed a reduction in the overall incidence of paediatric trauma presentation during the school closure period. This is comparable to the findings of other studies [4-8]. This reduction is likely due to several factors, such as the cancellation of organised team sports and the greater amount of time spent at home, leading to fewer opportunities for injury. Furthermore, parents may have treated minor injuries at home to minimise potential exposure to COVID-19. Moreover, as this study only measured referrals to our trauma and orthopaedic services, more patients with minor injuries may have been treated and discharged from the emergency department and general practitioner (GP) when previously they would have been referred for review.

Significantly fewer patients with comorbidities presented during school closure, suggesting that these children were more sheltered. Although children accounted for a smaller percentage of COVID-19 cases and had a slower rate of disease progression [9,10], most of the deaths or critical conditions requiring intensive care in the paediatric population occurred in those with underlying comorbidities [11,12].

Mechanism of injury

School closure had a significant effect on the patterns of mechanism of injuries children suffered, suggesting a change in social behaviours. In concordance with the findings of other studies on paediatric trauma presentation during the pandemic [13,14], we observed a significant decrease in sports-related injuries. Cessation of team sports would be the expected cause of this, thus proportionately affecting older-aged children more. We concur with Wild et al. [13] that the average age at presentation was lower during the lockdown period.

As children were confined to their homes and neighbourhoods, there was likely an increase in the use of bikes and scooters, which led to an increase in the rates of ride-on injuries. Studies worldwide reported that bicycle-related injuries in children increased substantially during the COVID-19 pandemic [13-17]. This highlights the importance of reinforcing bicycle safety education in children. However, this did not translate to higher rates of road traffic collision (RTC), with RTC presentations plummeting in lockdown. This was likely due to decreased traffic during that period. The UK parliament reported a 21% fall in road traffic in 2020 compared with 2019 [18]. The largest fall was during the first national lockdown when the overall traffic was 68% lower compared to the previous year [18].

An alarming finding was the significant increase in NAI concerns and subsequent presentations. School closures posed a huge burden on some families as more adults were made redundant or were working from home while caring for children out of school. One survey found that, during the lockdown period, parents were twice as likely to be furloughed (13.6%) than those without children (7.2%) [19]. Additionally, working parents with school-aged children said that their work had been affected by the pandemic and that 20% of this disruption was due to having to work around childcare responsibilities [19]. Increased financial and social stresses on family units may have led to environments that put more children at risk of NAI.

Anatomical location and type of injury

There was a proportional increase in upper limb injuries during school closure, which was likely due to the significant rise in ride-on injuries. Upper extremity injuries are recognised to be the most common site of trauma in bicycle-related injuries [20-23]. Although there was a reduction in the rate of lower limb injuries, the proportion of tibial shaft injuries rose significantly. Tibial fractures are the third most common paediatric long bone fractures after femur and forearm and account for 15% of all paediatric fractures [24]. Simple falls from play or free time activities are the leading cause of injury [25,26]; this is in line with our findings that there was an increase in the proportion of injuries associated with normal falls and outdoor toys during the school closure period. This may further explain the proportional rise in upper limb injuries as children have a habit of extending their arms to protect themselves when falling [27,28].

School closure saw a drop in patients presenting with less significant injuries such as sprains/wounds. We posit this is due to a combination of factors: overall fewer minor injuries due to reduced social activities, parents’ reluctance to attend local emergency departments for fear of exposing children to COVID-19 [29] and first-line emergency services managing and discharging minor injuries rather than referring on to the orthopaedic trauma team.

These findings reflect a clear shift in injury patterns in the paediatric population during the pandemic. Thus, improved guidelines for injury prevention are necessary for future social restrictions.

Management

The COVID-19 outbreak increased pressure on orthopaedic departments to manage more injuries conservatively. In line with updated guidelines set out by the British Orthopaedic Association and the British Society for Children’s Orthopaedic Surgery [30], this study found an increase in the use of conservative management during the school closure period. This may be due to limited availability of resources including personnel and operating theatre slots, reluctance to put patients and staff at increased risk of viral transmission and the responsibility to follow public health guidelines [31]. Long-term follow-up would be beneficial to evaluate the outcomes of reduced surgical management.

Follow-up

This study showed a rise in the rate of complications, but the absolute number of complications was lower. The higher complication rate from management may be skewed as there were more severe injuries seen at presentation and a reduction in patients presenting with less significant injuries. There was a significant increase in the re-presentation rate in the 2020 group. In 2019, the one case that re-presented was due to the recurrence of symptoms, and additional physiotherapy input was required. Reasons for representation in the lockdown cohort included ongoing symptoms of pain, instability and gait abnormalities. Despite the significant rise in re-presentation, only one case required additional intervention. These findings may be explained by the increase in parental stress and anxiety associated with the national lockdown [32].

This study observed an increase in the use of telephones to follow up with patients in the 2020 period; as life returns to normal, continued use of telemedicine would ensure fewer parents missing work and children missing school for clinical appointments. A systematic review evaluated the use of telemedicine service during the pandemic period in a wide range of specialities across the globe and showed that its application played a significant role in disease management, building a better healthcare system by avoiding depletion of medical resources, as well as protecting vulnerable patients [33]. In addition to the clinical efficiency, there may be an associated cost-benefit. A UK single-centre retrospective study evaluating changes in paediatric orthopaedic practice during lockdown found that there was a significant increase in telephone appointments and the use of self-removable casting technique and thus a reduction in in-person paediatric clinic appointments. This could potentially save £185,000 per annum [34]. These changes brought about by the lockdown have been demonstrated to be safe in the short term with no increase in complications demonstrated [34].

Limitations

A limitation of this study was that, firstly, all cases were identified retrospectively. The information collected may not be as robust as if data were collected prospectively.

Secondly, we only collected data from paediatric trauma cases that were referred and managed by our orthopaedic service. Some of the head injury cases would have been referred directly to neurosurgeons. Additionally, many paediatric trauma cases would have been managed by other specialities, such as general surgery, ear, nose and throat (ENT) or maxillofacial departments.

Thirdly, this study reflects the experience of the West Yorkshire Trauma Network, and it may not be possible to extrapolate this to paediatric trauma in the wider population during the pandemic period. Future extensions of this research would be multi-centre or nationwide to better understand the epidemiological trends.

Finally, part of the 12-month follow-up period for the 2019 group was during school closure. This may have an impact on the 2019 data. Some patients with minor complications may be less likely to present to the hospital during the pandemic.

## Conclusions

This regional retrospective cohort study observed a significant effect of the national COVID-19 lockdown on paediatric trauma presentation and management. There was a reduction in the overall volume of injuries, and the average age at presentation dropped by more than one year. There were decreased sports-related injuries and increased ride-on injuries. Although there was an increase in NAI concerns, suggestive of increased social pressures, the confirmed cases did not increase in absolute numbers. Finally, there was an increase in the use of conservative management and telemedicine. Further study is required to evaluate the long-term impacts on reduced surgical management.
